# Genotyping and subtyping of *Cryptosporidium* spp. and *Giardia duodenalis* isolates from two wild rodent species in Gansu Province, China

**DOI:** 10.1038/s41598-022-16196-1

**Published:** 2022-07-16

**Authors:** Jie Xu, Hua Liu, Yanyan Jiang, Huaiqi Jing, Jianping Cao, Jianhai Yin, Teng Li, Yeting Sun, Yujuan Shen, Xin Wang

**Affiliations:** 1 National Institute of Parasitic Diseases, Chinese Center for Disease Control and Prevention (Chinese Center for Tropical Diseases Research), Shanghai, 200025 China; 2 NHC Key Laboratory of Parasite and Vector Biology, Shanghai, 200025 China; 3 WHO Collaborating Centre for Tropical Diseases, Shanghai, 200025 China; 4 National Center for International Research on Tropical Diseases, Shanghai, 200025 China; 5 National Institute of Infectious Diseases, Chinese Center for Disease Control and Prevention, Beijing, 102206 China; 6 School of Global Health, Chinese Center for Tropical Diseases Research, Shanghai Jiao Tong University School of Medicine, Shanghai, 200025 China

**Keywords:** Microbiology, Molecular biology

## Abstract

*Cryptosporidium* spp. and *Giardia duodenalis* are commonly detected intestinal protozoa species in humans and animals, contributing to global gastroenteritis spread. The present study examined the prevalence and zoonotic potential of *Cryptosporidium* spp. and *G. duodenalis* in Himalayan marmots and Alashan ground squirrels in China's Qinghai-Tibetan Plateau area (QTPA) for the first time. Four hundred ninety-eight intestinal content samples were collected from five counties of QTPA of Gansu province, China. All samples were examined for *Cryptosporidium* spp. and *G. duodenalis* by PCR amplification. The resultant data were statistically analyzed by chi-square, Fisher's test and Bonferroni correction using SPSS software 25. 0. *Cryptosporidium* positive samples were further subtyped through analysis of the 60-kDa glycoprotein (*gp60*) gene sequence. A total of 11 and 8 samples were positive for *Cryptosporidium* spp. and *G. duodenalis*, respectively. Prevalence of *Cryptosporidium* spp. and *G. duodenalis* were 2.5% (10/399) and 1.5% (6/399) in Himalayan marmots, 1.0% (1/99) and 2.0% (2/99) in Alashan ground squirrels, respectively. Sequence analysis confirmed the presence of *C. rubeyi* (n = 2), ground squirrel genotype II (n = 7), chipmunk genotype V (n = 1) and horse genotype (n = 1). The horse genotype was further subtyped as novel subtype VIbA10. *G. duodenalis* zoonotic assemblages A (n = 1), B (n = 6), E (n = 1) were identified in the present study. This is the first study to identify *Cryptosporidium* spp. and *G. duodenalis* in Himalayan marmots and Alashan ground squirrels, suggesting the potential zoonotic transmission of the two pathogens in QTPA.

## Introduction

*Cryptosporidium* spp. and *Giardia duodenalis* are critical protozoan parasites responsible for diarrhea and infect a wide range of hosts including humans worldwide. Typically, contaminated food or water has been identified as the primary vehicle for *Cryptosporidium* spp. and *G. duodenalis* transmission^1,2^. Infection of these pathogens can also be acquired following contact with infected persons or animals directly^2,3^.

Currently, at least 45 valid *Cryptosporidium* spp. species and over 120 genotypes have been identified. Over 23 *Cryptosporidium* species/genotypes have been identified in humans, and *C. hominis* and *C. parvum* are the most common species (more than 90%) responsible for human cryptosporidiosis^4–12^. *G. duodenalis* is a complex protozoan species, and it has been divided into at least eight genetically different assemblages (A–H) based on genetic characterization. Among them, assemblages A and B are considered as critical zoonotic pathogens. Assemblages (C–H) are host-specific: assemblages C and D in canines, assemblage E in cloven-hoofed mammals, assemblage F in cats, assemblage G in rodents, and assemblage H in seals^13^. However, assemblages C, D, E and F have also been found in humans^14^.

Rodents can act as reservoirs or carriers for numerous zoonotic pathogens, including bacteria, parasites and viruses. Himalayan marmots (*Marmota himalayan*a) and Alashan ground squirrels (*Spermophilus alashanicus*) are two common wild rodent species distributed widely in Qinghai-Tibetan Plateau area (QTPA) of China. They typically reside near livestock, water sources and human environments. Among them, infected hosts can play essential roles in environmental contamination by excreting oocysts/cysts via feces^15^. Some epidemiological studies also revealed the identity of *Cryptosporidium* spp. and *G. duodenalis* in numerous investigated hosts in QTPA, such as wild Qinghai voles, plateau pikas, wild birds, cattle, yaks and sheep^16–20^. Furthermore, the zoonotic species and genotypes of *Cryptosporidium* spp. and *G. duodenalis* were also reported in environmental samples in QTPA, including sewage and river water, slaughterhouse water and vegetables from street markets^15,21^. However, no previously study about the prevalence and transmission of *Cryptosporidium* spp. and *G. duodenalis* in Himalayan marmots and Alashan ground squirrels in China was reported. In the present study, a cross-sectional investigation was carried out in Himalayan marmots and Alashan ground squirrels to understand the prevalence of *Cryptosporidium* spp. and *G. duodenalis* and assess the zoonotic potential at the genotype and subtype levels.

## Materials and methods

### Sample collection

**Figure 1:**
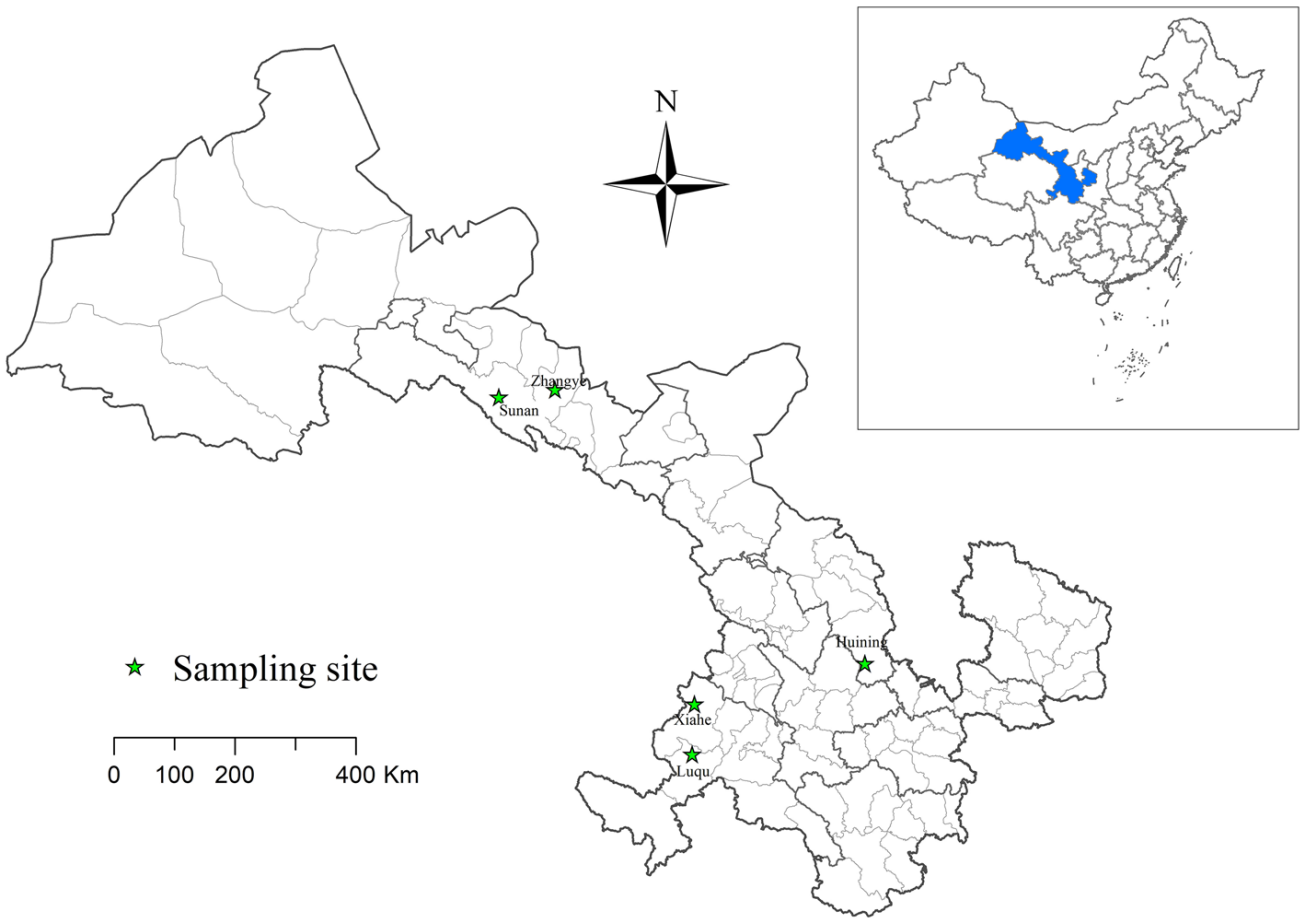
Distribution of five sampling sites from Gansu Province. The map was created with software ArcGIS version 10.0 (URL: https://www.esri.com).

During a period of three months from June to September 2017, 399 Himalayan marmots and 99 Alashan ground squirrels were captured live by mousetraps from QTPA of western China’s Gansu Province (Fig. 1), with the former from Luqu (n = 98), Sunan (n = 100), Xiahe (n = 102) and Zhangye (n = 99) and latter from Huining County (n = 99) (Table 1). These animals were euthanized with a high dose of CO_2_ following security measures. Intestinal content materials were directly collected from each animal in the local Center for Disease Control and Prevention (CDC) laboratory and placed in 2 ml sterile tubes. They were kept in a freezer and then transported in ice packs to our laboratory in Shanghai for further molecular analysis.

**Table 1:** Prevalence and molecular identification of *Cryptosporidium* spp. and *G. duodenalis* by rodent species and collection site.

Rodent species	Collection site	No. examined	*Cryptosporidium* spp.			*G. duodenalis*		
			No. positive (%)	Genotype (n)	Subtype (n)	No. positive (%)	Assemblage (n)	
				*SSU rRNA*	*gp60*		*gdh*	*bg*
Himalayan marmot (*Marmota himalayana*)	Luqu	98	0	–	–	0	–	–
	Sunan	100	7 (7.0)	*C. rubeyi* (1); ground squirrel genotype II (5); chipmunk genotype V (1)	–	0	–	–
	Xiahe	102	2 (2.0)	Ground squirrel genotype II (2)	–	3 (2.9)	B (1), E (1)	B (2), E (1)
	Zhangye	99	1 (1.0)	*C. rubeyi* (1)	–	3 (3.0)	B (1)	A (1), B (1)
Subtotal		399	10 (2.5)	*C. rubeyi* (2); ground squirrel genotype II (7); chipmunk genotype V (1)	–	6 (1.5)	B (2), E (1)	A (1), B (3), E (1)
Alashan ground squirrel (*Spermophilus alaschanicus*)	Huining	99	1 (1.0)	Horse genotype (1)	VIbA10^a^(1)	2 (2.0)	B (2)	B (2)
Total		498	11 (2.2)	*C. rubeyi* (2); ground squirrel genotype II (7); chipmunk genotype V (1); horse genotype (1)	VIbA10^a^(1)	8 (1.6)	B (4), E (1)	A (1), B (5), E (1)

^a^Novel subtype.

### DNA extraction

Genomic DNA was extracted using the DNeasy Blood & Tissue Kit (Cat. #69506; Qiagen, Hilden, Germany) according to the manufacturer’s instructions. Extracted DNA was stored at − 20 °C in a freezer until further use.

### PCR amplification

*Cryptosporidium* spp. was detected by nested PCR amplification of the fragment (approximately 830 bp) of the small subunit (*SSU*) *rRNA* gene^22^. Subtyping of *Cryptosporidium* spp. was performed by sequence analysis of the 60 kDa glycoprotein (*gp60*) gene^23^. All the isolates of *Cryptosporidium*-positive samples were selected for further sequence characterization via the *actin* gene and 70-kDa heat shock protein (*HSP70*) gene^61,62^. The assemblages of *G. duodenalis* were identified and subtyped by amplifying the β-giardin (*bg*), glutamate dehydrogenase (*gdh*) and triosephosphate isomerase (*tpi*)^24–26^. DNA of human-derived *C. parvum* and *C. viatorum* were used as positive controls in PCR tests to amplify the *SSU rRNA*, *gp60*, *actin* and *HSP70* genes, respectively. Premiers and reaction conditions were shown in Supplementary Table S1. DNA of human-derived *G. duodenalis* was used as a positive control in PCR tests to amplify the *bg*, *gdh* and *tpi* genes. DNase-free water was used as a negative control in each PCR test. The secondary PCR products were visualized under UV light after electrophoresis on a 1.5% agarose gel containing GelRed (Biotium Inc., Hayward, CA, USA).

### Nucleotide analysis

All secondary PCR amplicons of the expected size were sequenced on ABI 3730 DNA Analyzer (Applied Biosystems, Foster City, USA) and Big Dye Terminator v3.1 Cycle Sequencing Kit (Applied Biosystems). Sequence accuracy was confirmed by bi-directional sequencing of all the PCR-positive products. Obtained DNA sequences were aligned with reference sequences deposited in GenBank databases (http://www.ncbi.nlm.nih.gov) using Clustal X (http://www.clustal.org/) to determine the species/subtypes of *Cryptosporidium* spp. and assemblages of *G. duodenalis*. Phylogenetic analyses at the *SSU rRNA*, *actin*, *HSP70* and *gp60* gene loci were performed using the neighbor-joining model in MEGA 11 (http://www.megasoftware.net). Bootstrap analysis was used to assess the robustness of the clusters using 1 000 replicates.

### Statistical analysis

Differences in prevalence of *Cryptosporidium* spp. and *G. duodenalis* in Himalayan marmots and Alashan ground squirrels were compared among species and investigated area using the were processed with chi-square test, Fisher's exact test and pairwise comparisons used a Bonferroni correction to control for multiple testing. All the statistical analyses were performed using SPSS 25. 0 (SPSS Inc., New York, USA). Differences were considered significant at P < 0.05.

### Ethics statements

This study was performed in strict accordance with the recommendations in the Guide for the Care and Use of Laboratory Animals of the National Institute of Parasitic Diseases, Chinese Center for Disease Control and Prevention. The protocol was approved by the Laboratory Animal Welfare & Ethics Committee (LAWEC), National Institute of Parasitic Diseases, Chinese Center for Disease Control and Prevention (Permit Number: NIPD-2016-15).

## Results

### Prevalence of *Cryptosporidium* spp. and *G. duodenalis*

Using PCR amplification and sequence analysis, *Cryptosporidium* spp. and *G. duodenalis* were found in Himalayan marmots and Alashan ground squirrels. The agarose gel electrophoresis results of PCR amplification products were shown in Supplementary Fig. S1 (partial samples) and Fig. S2 (partial samples). A total of 11 and 8 samples were positive for *Cryptosporidium* spp. and *G. duodenalis*, respectively. Prevalence of *Cryptosporidium* spp. and *G. duodenalis* were 2.5% (10/399) and 1.5% (6/399) in Himalayan marmots, and 1.0% (1/99) and 2.0% (2/99) in Alashan ground squirrels, respectively (Table 1). The statistical analysis showed no significant difference in the prevalence of *Cryptosporidium* spp. (*P* = 0.365) and *G. duodenalis* (*P* = 0. 714) between Himalayan marmots and Alashan ground squirrels. Different prevalence of *Cryptosporidium* spp. and *G. duodenalis* were observed in five different investigated areas (Table 1): Luqu (0.0% and 0.0%), Sunan (7.0% and 0.0%), Xiahe (2.0% and 2.9%), Zhangye (1.0% and 3.0%) and Huining (1.0% and 2.0%). Moreover, there was no significant difference observed in the prevalence of *Cryptosporidium* spp. and *G. duodenalis* in each paired comparison between investigated areas (*P* > 0.05). No mixed infection of *Cryptosporidium* spp. and *G. duodenalis* identified in this study.

### *Cryptosporidium* genotypes and subtypes

Based on sequence analysis of the *SSU rRNA* gene, a total four species/genotypes of *Cryptosporidium* spp. were identified out of 11 isolates, including *C. rubeyi* (n = 2), ground squirrel genotype II (n = 7), and chipmunk genotype V (n = 1) in Himalayan marmots, and horse genotype (n = 1) in Alashan ground squirrels. *Cryptosporidium* ground squirrel genotype II was dominant in Himalayan marmots, accounting for 70.0% (7/10) of *Cryptosporidium* isolates. At the *SSU rRNA* gene locus, the two identical sequences of *C. rubeyi* shared the most significant identity (98.43%) with that of *C. rubeyi* (DQ295012) from California ground squirrels in the USA, with 13 base differences. Seven sequences of ground squirrel genotype II were identical and shared the most prominent similarity (98.28%) to that of the ground squirrel genotype II (KT027480) from black-tailed prairie dogs, with 14 base differences. The sequence of the chipmunk genotype V had 98.90% homology with that (MW521250) of the chipmunk genotype V from chinchillas in China, with nine base differences. The sequence of the horse genotype obtained in the present study had 100% homology with a sequence (MK775040) from a horse in China. The horse genotype isolate was further subtyped by sequence analysis of the *gp60* gene. This subtype belonged to the VIb subtype family and was identified as VIbA10 (GenBank: MW531716).

None of the two sequences of *C. rubeyi* were successfully amplified at the *HSP70* gene locus but successfully amplified at the *actin* gene locus, and the two sequences were identical to each other, had 100% similarity with that of *C. rubeyi* (GenBank: KT027530) from black-tailed prairie dog. Meanwhile, two of seven isolates of ground squirrel genotype II were successfully amplified at the *actin* gene locus, and the two isolates shared the same sequence which had 97.68% similarity with that of ground squirrel genotype II (GenBank: KT027545) from black-tailed prairie dog in the USA. The *HSP70* sequences have not been reported for ground squirrel genotype II. Three of seven isolates of ground squirrel genotype II were successfully amplified at the *HSP70* gene locus and had 93.50% similarity with that of *C. viatorum* (GenBank: JX978274) from human in Guatemala. The sequence of chipmunk genotype V was only successfully amplified at the *actin* gene locus and shared 99.69% identity with that of chipmunk genotype V (MW521262) from chinchillas in China. Horse genotype was successfully amplified at the *actin* gene locus and shared 100% similarity with horse genotype (KU892571) isolated from humans of Kenya.

**Figure 2:**
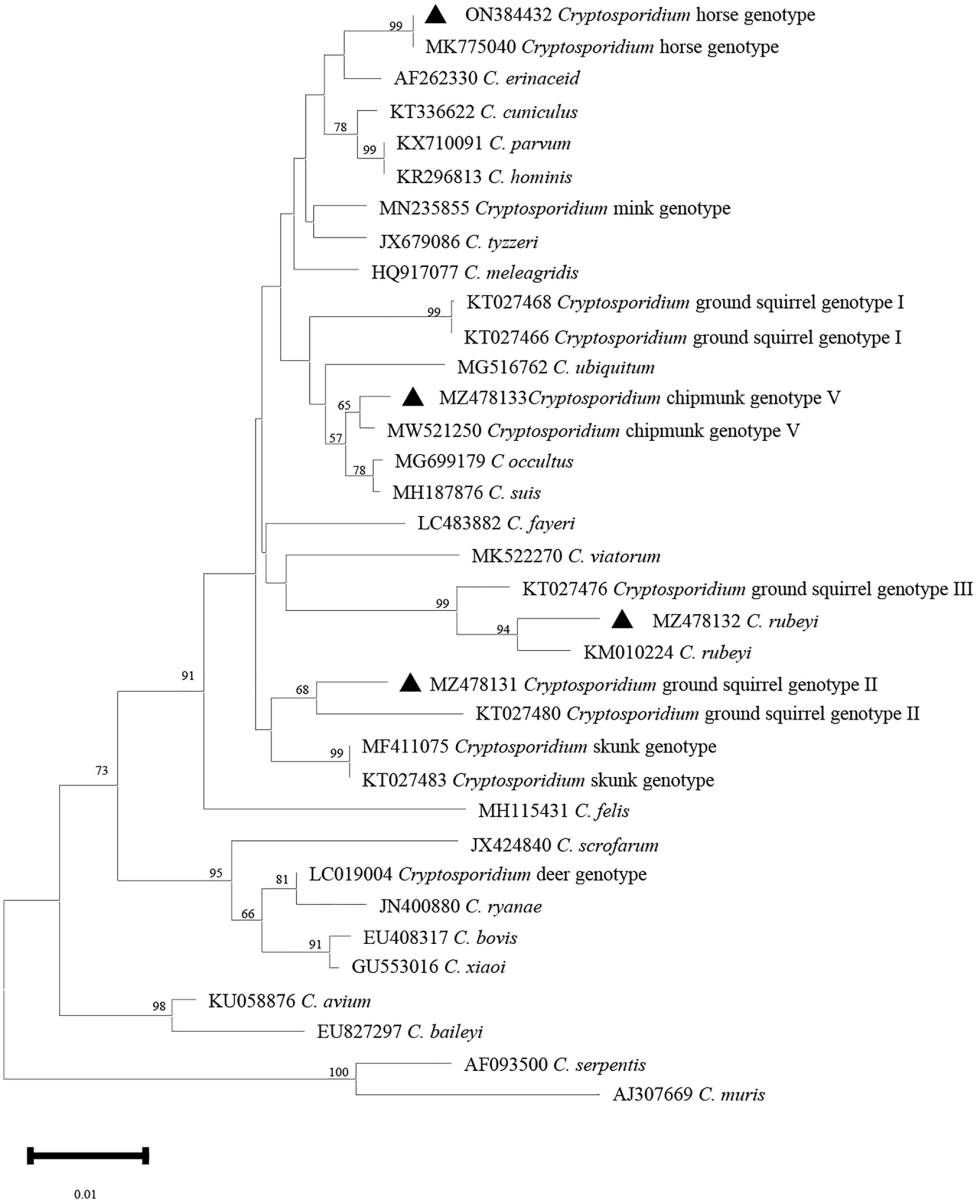
Phylogenetic relationship among *Cryptosporidium* spp. based on a neighbor-joining tree of the *SSU rRNA* gene. The numbers on the branches are percent bootstrapping values from 1000 replicates, and the sequences generated in the present study are indicated with the triangles.

**Figure 3:**
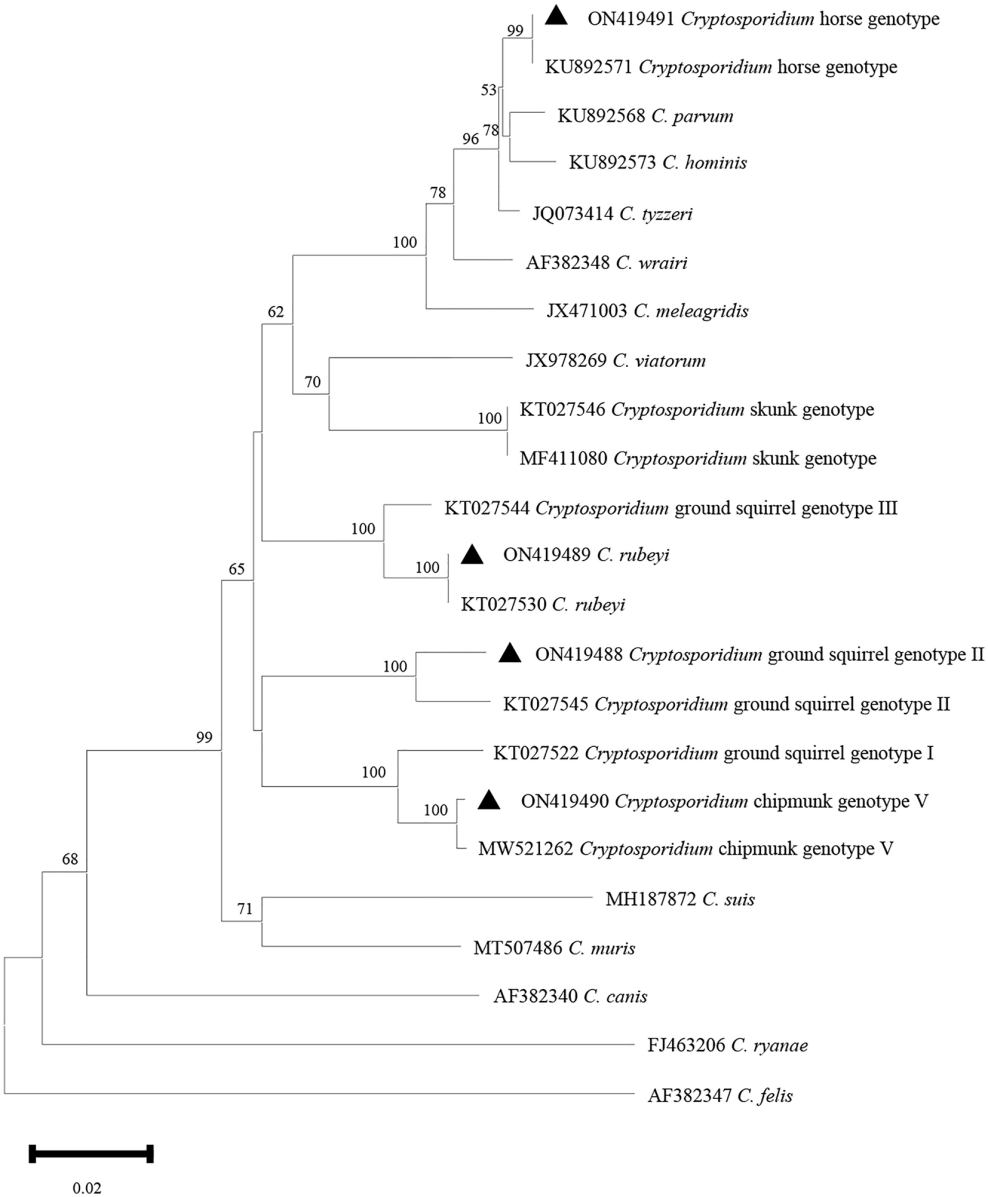
Phylogenetic relationship among *Cryptosporidium* spp. based on a neighbor-joining tree of the *actin* gene. The numbers on the branches are percent bootstrapping values from 1000 replicates, and the sequences generated in the present study are indicated with the triangles.

**Figure 4:**
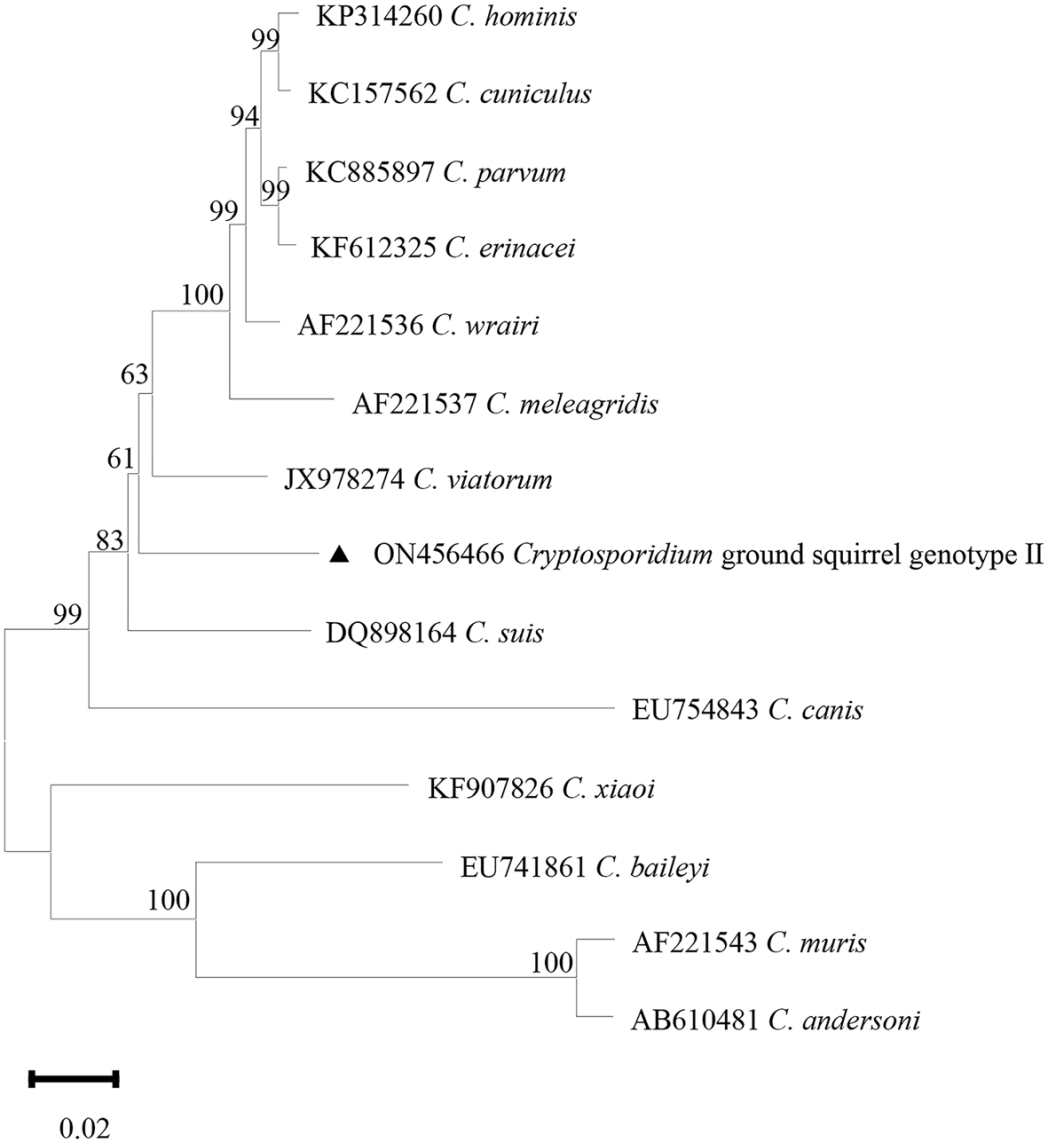
Phylogenetic relationship among *Cryptosporidium* spp. based on a neighbor-joining tree of the *HSP70* gene. The numbers on the branches are percent bootstrapping values from 1000 replicates, and the sequences generated in the present study are indicated with the triangles.

**Figure 5:**
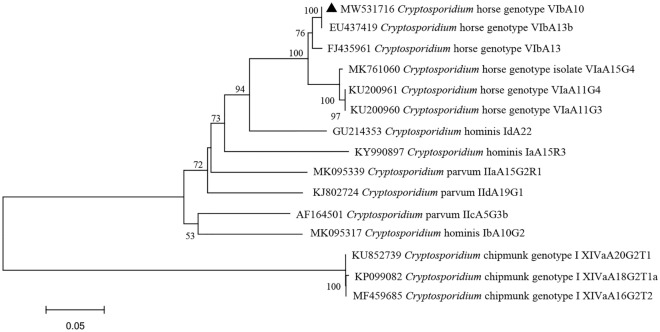
Phylogenetic relationship of *Cryptosporidium* subtypes based on a neighbor-joining tree of the *gp60* gene. The numbers on the branches are percent bootstrapping values from 1000 replicates, and the sequences generated in the present study are indicated with the triangles.

Phylogenetic analyses of the *SSU rDNA*, *actin*, *HSP70* and *gp60* gene sequences were shown in Figs. 2, 3, 4 and 5.

### *G. duodenalis* assemblages

A total of eight *G. duodenalis* isolates were amplified and sequenced successfully in Himalayan marmots and Alashan ground squirrels in this study. Assemblages A, B and E were identified in one, four and one Himalayan marmot samples, respectively. Assemblage B was found in two Alashan ground squirrel samples. Meanwhile, assemblage B was observed to show a predominance (75.0%, 6/8) in the detected animals. The *gdh* and *bg* genes were successfully amplified in five samples—assemblages B (n = 4) and E (n = 1) and seven samples—assemblages A (n = 1), B (n = 5) and E (n = 1), respectively (Table 1). In this study, PCR amplification failed at the *tpi* locus.

At the *gdh* locus, two assemblage B sequences had 100% homology with beaver-derived assemblage B isolated (KM977648) from China. Another two different assemblage B sequences were 100% identical to golden monkey-derived assemblage B isolate (MK952602) from China, and one assemblage E sequence was 100% identical to a pig-derived assemblage E isolate (MK426742) from South Korea. At the *bg* locus, five assemblage B sequences shared 100% homology with squirrel monkey-derived assemblage B isolate (KJ888974) from China, one assemblage A sequence had 100% homology with human-derived assemblage A isolates (GQ329671) from Sweden and chipmunk-derived isolate (MF671918) from China, one assemblage E sequence (GenBank: MZ494459) shared the most considerable similarity (99.79%) to that (KY633473) from a Tibetan sheep in China, with only one base difference.

## Discussion

In this study, the overall prevalence of *Cryptosporidium* spp. were 2.2% (11/498), with 2.5% in Himalayan marmots, and 1.0% in Alashan ground squirrels. There was no significant difference in the prevalence of *Cryptosporidium* spp. and *G. duodenalis*, and we will enlarge the research sample size for further verification. Other studies reported much higher prevalence of *Cryptosporidium* spp. in wild rodent species in China than this study, including in house mice (3.2%, 1/31), long-tailed rats (3.6%, 4/111 and 55.3%, 21/38), brown rats (6.3%, 4/64; 9.1%, 22/242 and 28.6%, 16/56), wild plateau pikas (6.3%, 4/64), Qinghai voles (8.9%, 8/90), Asian house rats (18.0%, 21/117; 18.2%, 6/33 and 73.9%, 4/46), Brandt’s voles (18.7%, 127/678), Muridae (40.0%, 4/10)^20,27–32^. The prevalence in this study was also lower than that in some pet rodent species, including in bamboo rats (3.3%, 3/92), Siberian hamsters (7.8%, 4/51), red squirrels (8.6%, 27/314 and 26.3%, 5/19), chinchillas (9.3%, 26/280 and 10.0%, 14/140), campbell hamsters (10.0%, 3/30 and 22.2%, 6/27), Siberian chipmunks (30.0%, 6/20), gold hamsters (32.0%, 16/50), chipmunks (50.0%, 1/2 and 75.0%, 3/4), guinea pigs (52.3%, 162/310 and 85.0%, 34/40), Roborovski dwarf hamsters (100.0%,1/1)*,* and higher than that in pet red-bellied tree squirrels (1.4%, 4/287)^29,33–38^. In addition, there was difference between prevalence in different farmed and laboratory rodent species, including farmed bamboo rats (2.1%, 9/435 and 29.5%, 209/709), farmed brown rats (7.1%, 12/168), experimental brown rats (0.6%, 2/355), laboratory mice (1.7%, 4/229), laboratory rats (4.0%, 1/25)^27,29,39–41^. These variations in the prevalence of *Cryptosporidium* spp. in different studies may be explained by many factors, including the population densities, the health status of hosts, management systems, experimental methods and source region^42^.

To date, including *Cryptosporidium* species/genotypes obtained in this study, a total of 14 *Cryptosporidium* species and 17 genotypes have been detected in 16 studies of various rodents in China (Table 2)^20,27–37,39–41^. Among them, 11 species/genotypes have been detected in humans: *C. parvum*, *C. muris*, *C. ubiquitum*, *C. andersoni*, *C. occultus*, *C. viatorum*, *C. canis*, *C. suis*, *C. erinaceid*, *C. tyzzeri* and horse genotype^4^, indicating rodents may play essential roles in the transmission of zoonotic cryptosporidiosis.

**Table 2:** *Cryptosporidium* species/genotypes in rodents in China.

Host species (Latin name)	No. positive (%)	Species/genotype (n)	Sample source	References
Alashan ground squirrels (*Spermophilus alaschanicus*)	1/99 (1.0)	Horse genotype (1)	Wild	This study
Asian house rats (*Rattus tanezumi*)	6/33 (18.2)	*C. parvum* (3), *C. muris* (3)	Wild	^27^
Asian house rats (*Rattus tanezumi*)	6/33 (18.2)	*C. tyzzer* (1), rat genotype II (1), rat genotype III (1), *C. tyzzer* + rat genotype II (1), *C. tyzzer* + rat genotype III (1)	Wild	^29^
Asian house rats (*Rattus tanezumi*)	34/46 (73.9)	Rat genotype IV (24), rat genotype III (8), *C. occultus* (1), *C. erinacei* (1)	Wild	^32^
Bamboo rats (*Rhizomys sinensis*)	9/435 (2.1)	Bamboo rat genotype I (5), *C. parvum* (2), *C. occultus* (1), bamboo rat genotype II (1)	Farmed	^40^
Bamboo rats (*Rhizomys sinensis*)	3/92 (3.3)	*C. parvum* (3)	Pet	^34^
Bamboo rats (*Rhizomys sinensis*)	209/709 (29.5)	*C. ubiquitum*-like (85), *C. parvum* (78), *C. parvum*-like (45), *C. occultus* (1),	Farmed	^41^
Brandt's voles (*Lasiopodomys brandtii*)	127/678 (18.7)	*C. suis*, muskrat genotype II, Brandt's voles genotype I	Wild	^31^
Brown rats (*Rattus norvegicus*)	4/64 (6.3)	*C. tyzzer* (3), *C. tyzzer* + rat genotype III (1)	Wild	^29^
Brown rats (*Rattus norvegicus*)	12/168 (7.1)	*C. parvum* (9), *C. muris* (3)	Farmed	^27^
Brown rats (*Rattus norvegicus*)	22/242 (9.1)	*C. ratti* (14), rat genotype IV (6), *C. occultus* (1)	Wild	^30^
Brown rats (*Rattus norvegicus*)	16/56 (28.6)	Rat genotype IV (13), *C. muris* (1), *C. occultus* (1), rat genotype III (1)	Wild	^32^
Campbell hamsters (*Phodopus campbelli*)	3/30 (10.0)	*C. parvum* (1), *C. andersoni* (1), *C. muris* + *C. parvum* (1)	Pet	^29^
Campbell hamsters (*Phodopus campbelli*)	6/27 (22.2)	Hamster genotype (4), *C. andersoni* (2)	Pet	^38^
Chichillas (*Chinchilla lanigera*)	26/280 (9.3)	*C. ubiquitum* (23), *C. parvum* (2), chipmunk genotype V (1)	Pet	^38^
Chipmunks (*Eutamias asiaticus*)	1/2 (50.0)	Ferret genotype (1)	Pet	^37^
Chipmunks (*Eutamias asiaticus*)	3/4 (75.0)	Ferret genotype (2), chipmunk genotype V (1)	Pet	^38^
Edward's long-tailed rats (*Leopoldamys edwardsi*)	21/38 (55.3)	Rat genotype IV (13), rat genotype III (1), *C. muris* (1), *C. occultus* (1)	Wild	^32^
Experimental brown rats (*Ruttus norvegicus*)	2/355 (0.6)	*C. ubiquitum* (1), undetermined *Cryptosporidium* genotype (1)	Laboratory	^39^
Gold hamsters (*Mesocricetu auratus*)	16/50(32.0)	*C. muris* (6), *C. andersoni* (5), *C. parvum* (2), *C. muris* + *C. parvum* (1), *C. andersoni* + *C. parvum* (1)	Pet	^29^
Guinea pigs (*Cavia porcellus*)	162/310 (52.3)	*C. wrairi* (129), *C. homai* (32), *C. muris* (1)	Pet	^38^
Guinea pigs (*Cavia porcellus*)	34/40 (85.0)	*C. wrairi* (30)	Pet	^29^
Himalayan marmots (*Marmota himalayana*)	10/399 (2.5)	*C. rubeyi* (2), ground squirrel genotype II (7), chipmunk genotype V (1)	Wild	This study
House mice (*Mus musculus*)	1/31 (3.2)	*C. muris* (1)	Wild	^27^
Laboratory mice (*Mus musculus*)	4/229 (1.7)	*C. tyzzer* (4)	Laboratory	^29^
Laboratory rats (*Rattus norvegicus*)	1/25 (4.0)	*C. tyzzer* (1)	Laboratory	^29^
Long-tailed rats (*Leopoldamys edwardsi*)	4/111 (3.6)	*C. viatorum* (4)	Wild	^28^
Muridae (*Niviventer fulvescens*)	4/10 (40.0)	Rat genotype III (2), rat genotype IV (2)	Wild	^32^
Pet chinchillas (*Chinchilla lanigera*)	14/140 (10.0)	*C. ubiquitum* (13), *C. parvum* (1)	Pet	^36^
Qinghai voles (*Microtus fuscus*)	8/90 (8.9)	*C. parvum* (3), Qinghai vole genotype (3), *C. canis* (1), *C. ubiquitum* (1)	Wild	^20^
Red-bellied tree squirrels (*Callosciurus erythraeus*)	4/287 (1.4)	Rat genotype II (2), *C. parvum* (1), *C. wrairi* (1)	Pet	^33^
Red squirrels (*Sciurus vulgaris*)	27/314 (8.6)	rat genotype II (8), ferret genotype (8), chipmunk genotype III (5), *C. ratti* (4), C. parvum (2)	Pet	^35^
Red squirrel (*Sciurus vulgaris*)	5/19 (26.3)	Ferret genotype (5)	Pet	^29^
Roborovski dwarf hamsters (*Phodopus roborovskii*)	1/1 (100)	*C. muris (1)*	Pet	^38^
Siberian chipmunks (*Tamias sibiricus*)	6/20 (30.0)	Ferret genotype (3), ferret genotype + *C. parvum* (1), *C. muris* + *C. parvum* + chipmunk genotype III (1)	Pet	^29^
Siberian flying squirrels (*Pteromys volans*)	1/1 (100)	*C. ubiquitum* (1)	Pet	^38^
Siberian hamsters (*Phodopus sungorus*)	4/51 (7.8)	*C. muris* (1), *C. parvum* (1), *C. andersoni* + *C. parvum* (1), hamster genotype (1)	Pet	^29^
Siberian hamsters (*Phodopus sungorus*)	32/37 (86.5)	Hamster genotype (26),*C. andersoni* (6)	Pet	^38^
Syrian hamsters (*Mesocricetus auratus)*	26/30 (86.7)	*C. andersoni* (26)	Pet	^38^
White-toothed rats *(Berylmys bowersi)*	21/117 (18.0)	*C. viatorum* (21)	Wild	^28^
Wild plateau pikas (*Ochotona curzoniae*)	4/64 (6.3)	*C. parvum* (2), pika genotype (2)	Wild	^20^

Plus signs indicate that the sample was co-infected with different *Cryptosporidium* species/genotypes.

Altogether, four *Cryptosporidium* species/genotypes were identified in this study: *C. rubeyi*, ground squirrel genotype II, chipmunk genotype V in Himalayan marmots, and horse genotype in Alashan ground squirrels. *C. rubeyi* was characterized by numerous wild rodent hosts such as golden-mantled ground squirrels, California ground squirrels, Belding's ground squirrels, and black-tailed prairie dogs^43,44^. Previously ground squirrel genotype II and chipmunk genotype V were only identified in black-tailed prairie dogs in the USA^43^ and chinchillas in China^38^, respectively. Our identification of ground squirrel genotype II and chipmunk genotype V expanded the host range of the two genotypes. Horse genotype was initially isolated from a Przewalski wild horse at the Prague Zoo in the Czech Republic, and commonly detected in horses and donkeys, occasionally found in neonatal calves and hedgehogs^45,46^. Horse genotype has also been found in human patients with diarrhea in the UK and the USA, suggesting its zoonotic potential^47–49^. In the present study, the horse genotype was identified in rodents for the first time, indicating it has a broader range of host than initially anticipated. Horse genotype isolated from Alashan ground squirrels was further identified as novel subtype VIbA10. Currently, two subtype families are recognized within the *Cryptosporidium* horse genotype by sequence analysis targeting the *gp60* gene: the VIa subtype family in animals (horses, donkeys and calves, etc.) and the VIb subtype family in humans and hedgehogs.

The present study detected the infection of *Cryptosporidium* spp. in wild rodent species of the genus *Marmota* and genus *Spermophilus.* Further, eight previous studies have reported the occurrence of *Cryptosporidium* species/genotypes in other three species of the genus *Marmota* and other four species of genus *Spermophilus*: including *C. ubiquitum* in woodchucks (*Marmota monax*) in the USA^50,51^; *C. parvum* in yellow-bellied marmots (*Marmota flaviventris*) in the USA^52^; *C. andersoni* in Bobak marmots (*Marmota bobac*) in the Czech Republic^45^; *C. rubeyi* in California ground squirrels (*Spermophilus beecheyi*) in the USA, Belding's ground squirrels (*Spermophilus beldingi*) and golden-mantled ground squirrels (*Spermophilus lateralis*) in the USA^44,53,54^; ground squirrel genotype I and ground squirrel genotype III in thirteen-lined ground squirrels (*Spermophilus tridecemlineatus*) in USA^43^.

In this study, the overall prevalence of *G. duodenalis* were 1.6% (8/498), with 1.5% (6/399) for Himalayan marmots and 2.0% (2/99) for Alashan ground squirrels. This study reported much lower prevalence of *G. duodenalis* than other studies in wild rodent species in China: house mouse (3.2%, 1/31); Asian house rat (6.1%, 2/33); brown rat (6.6%, 11/168 and 9.3%, 33/355); pet chipmunks (8.6%, 24/279); bamboo rat (10.8%, 52/480); coypus (12.3%, 38/308); pet chinchillas (27.1%, 38/140)^27,39,55–58^ (Table 3).

**Table 3:** *G. duodenalis* assemblages in rodents in China.

Host species (Latin name)	No. positive (%)	Assemblages (n)			Sample source	References
		*bg*	*gdh*	*tpi*		
Alashan ground squirrels (*Spermophilus alashanicus*)	2/99 (2.0)	B (2)	B (2)		Wild	This study
Asian house rats (*Rattus tanezumi*)	2/33 (6.1)	G (2)	G (1)	G (1)	Wild	^27^
Bamboo rats (*Rhizomys sinensis*)	52/480 (10.8)	B (52)	B (27)	B (12)	Farmed	^56^
Brown rats (*Rattus norvegicus*)	11/168 (6·6)	G (11)	G (9)	G (10)	Wild	^27^
Brown rats (*Ruttus norvegicus*)	33/355 (9.3)	G (19)	G (20)	G (21)	Laboratory	^39^
Coypus (*Myocastor coypus*)	38/308 (12.3)	B (11), A (1)	B (10), A (1)	B (22), A (3)	Farm	^58^
Himalayan marmots (*Marmota himalayan*a)	6/399 (1.5)	A (1), B (3), E (1)	B (2), E (1)	–	Wild	This study
House mice (*Mus musculus*)	1/31 (3.2)	G (1)	–	G (1)	Wild	^27^
Pet chinchillas (*Chinchilla lanigera*)	38/140 (27.1)	A (4), B (8)	A (4), B (16)	A (3), B (3)	Pet	^57^
Pet chipmunks (*Eutamias asiaticus*)	24/279 (8.6)	G (11), A (13)	G (7), A (10)	G (4), A (13)	Pet	^55^

In this study, the sequences of amplicons from *G. duodenalis*-positive samples were determined to be assemblages A, B, and E, with assemblages B showing dominance in the detected animals. Assemblages A, B and E were identified in Himalayan marmots and assemblage B in Alashan ground squirrels. *G. duodenalis* assemblages in Himalayan marmots were richer than Alashan ground squirrels. As we know, in previous studies, *G. duodenalis* infections of Chinese rodents were reported to be caused by assemblages A, B and G^27,39,55–58^. Among them, assemblages A and B have a broad host range and are commonly found in humans^56^. Some recent studies in China also reported the occurrence of assemblage A in pet chipmunks, coypus and pet chinchillas, while assemblage B in bamboo rats, coypus and pet chinchillas^55–58^. These two assemblages were detected in this study suggest that Himalayan marmots and Alashan ground squirrels can play roles in the zoonotic dissemination of *G. duodenalis.* Assemblage E is commonly found in a range of hoofed livestocks and occasionally found in rodent species, and it has also been found in human cases, indicating that this assemblage is of zoonotic significance^59,60^.

In the investigated areas of QTPA, wild rodent species Himalayan marmots and Alashan ground squirrels have strong migration habits and often share pasture with humans, herbivorous animals and other wild animals. Results of this study suggest that these two wild rodent species may play a role in the transmission cycle of *Cryptosporidium* spp. oocysts and *G. duodenalis* cysts among humans, animals, water sources and fresh produce in QTPA grassland ecosystem.

## Conclusion

This study examined the prevalence and zoonotic potential of *Cryptosporidium* spp. and *G. duodenalis* in Himalayan marmots and Alashan ground squirrels in the Qinghai-Tibetan Plateau area (QTPA) of China for the first time. Four *Cryptosporidium* species/genotypes were identified, including *C. rubeyi*, ground squirrel genotype II, chipmunk genotype V and horse genotype (novel subtype VIbA10). These two rodent species identified *G. duodenalis* zoonotic assemblages A, B, and E. The results expanded the host range of *Cryptosporidium* spp. and *G. duodenalis*, providing more information on the prevalence, epidemiology and genetic characterizations of the two pathogens in Himalayan marmots and Alashan ground squirrels. Further surveys are also required to understand the prevalence and transmission dynamics of the two pathogens.

## Supplementary Information

Supplementary Information 1.

Supplementary Information 2.

Supplementary Information 3.

Supplementary Information 4.

## Data Availability

Nucleotide sequences of this article were deposited in the GenBank database under following accession numbers: MZ478131-MZ478133, ON384432 (*SSU rRNA*), ON419488-ON419491 (*actin*), ON456466 (*HSP70*), MW531716 (*gp60*) for *Cryptosporidium* spp*.*; MZ494459 (*bg*) for *G. duodenalis*.
