# Supplementary figures and images for: Genotyping and subtyping of Cryptosporidium spp. and Giardia duodenalis isolates from two wild rodent species in Gansu Province, China
Source: Sci Rep. 2022 Jul 16;12:12178. doi: 10.1038/s41598-022-16196-1 (PMC9288474; doi:10.1038/s41598-022-16196-1)

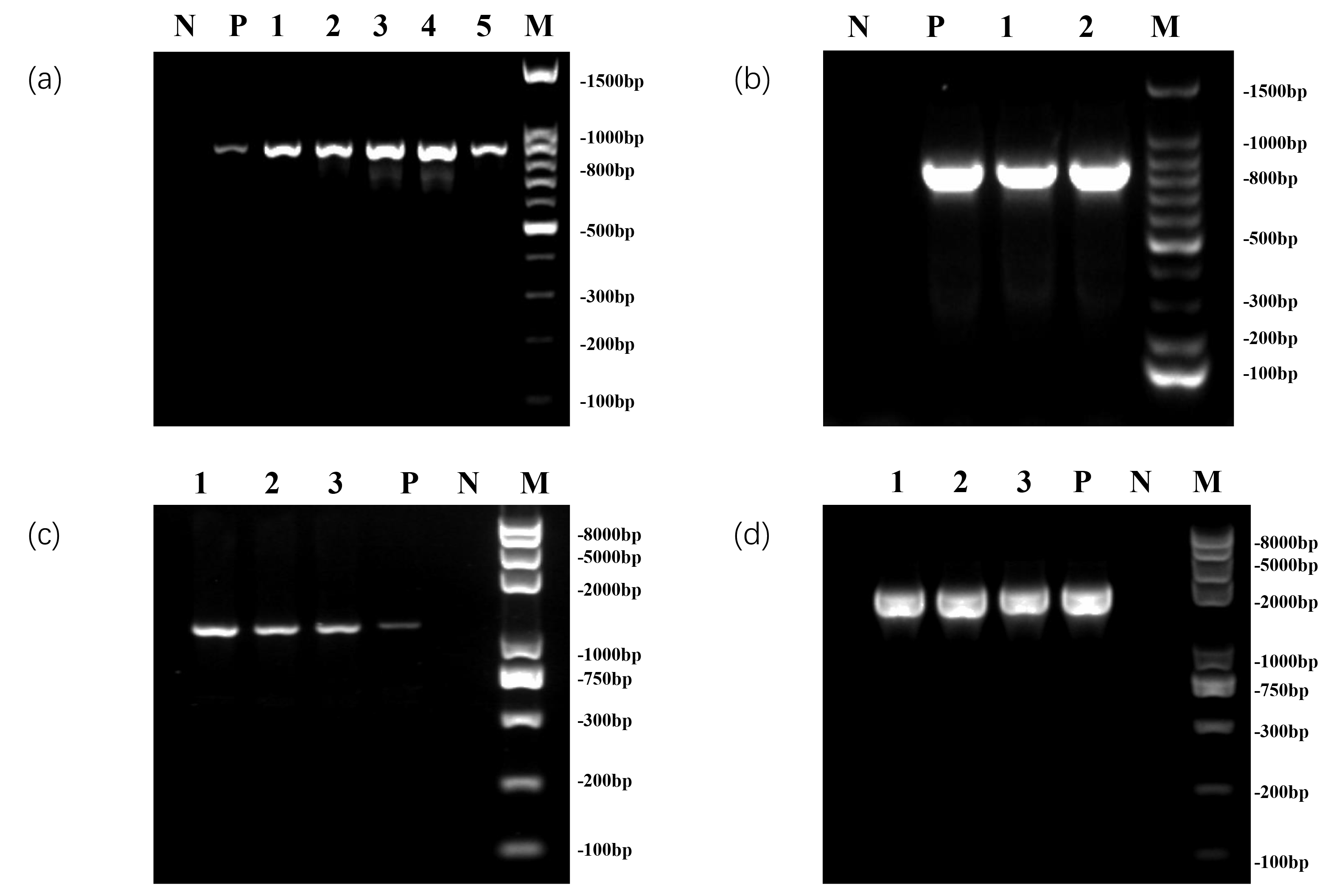

Supplement: Supplementary file 1 — Supplementary Information 1. [file 41598_2022_16196_MOESM1_ESM.tif]

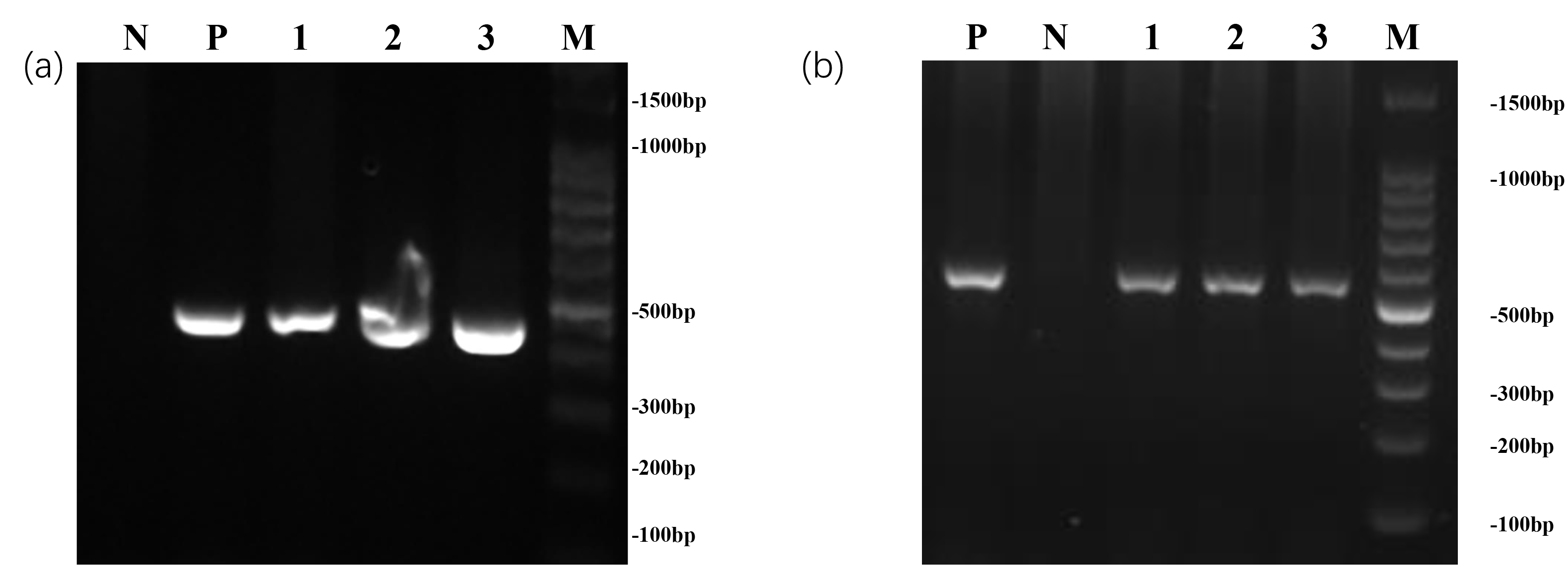

Supplement: Supplementary file 2 — Supplementary Information 2. [file 41598_2022_16196_MOESM2_ESM.tif]

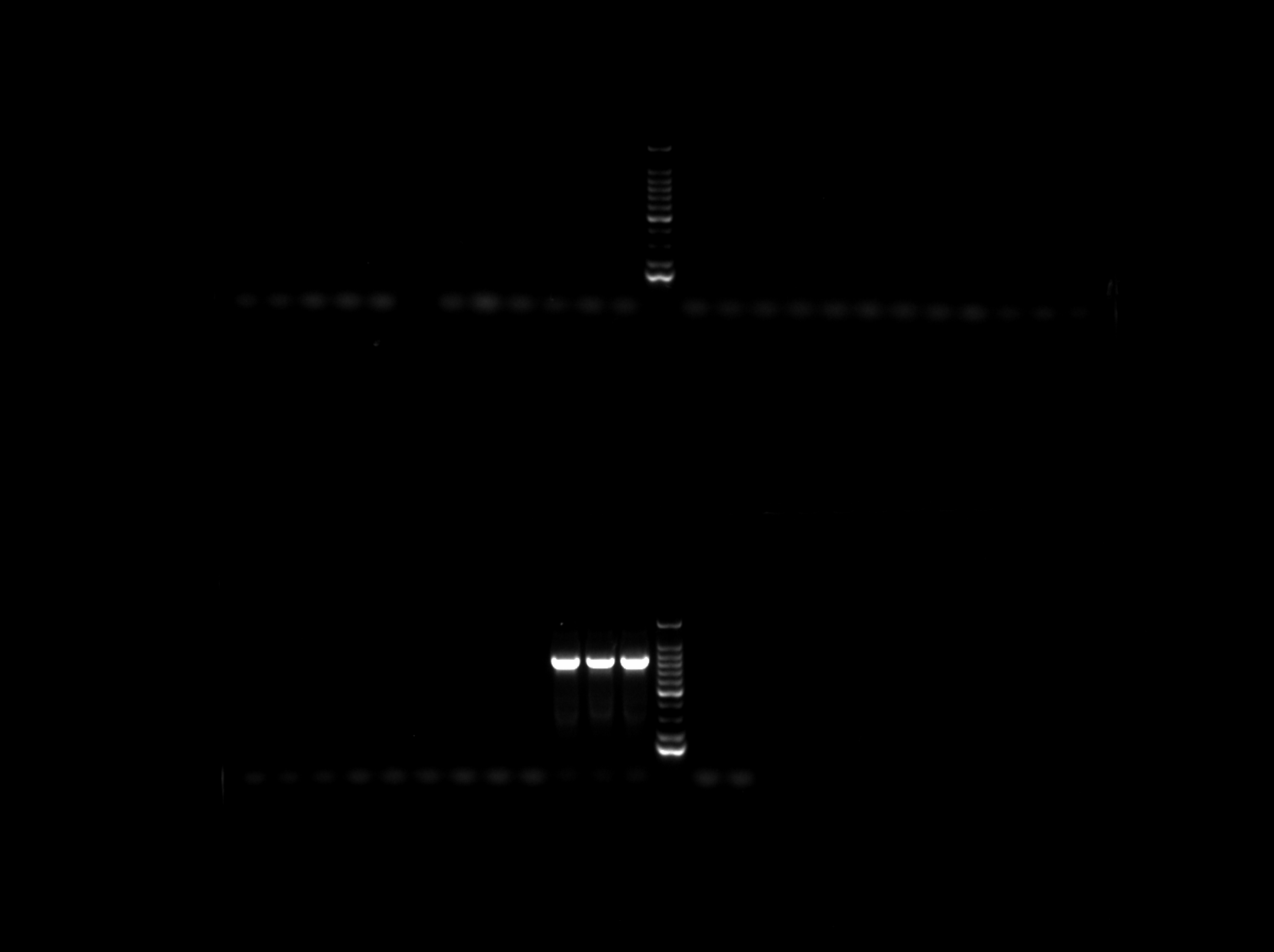

Supplement: Supplementary file 4 — Supplementary Information 4. [file 41598_2022_16196_MOESM4_ESM.zip › Cryptosporidium gp60 gene.tif]

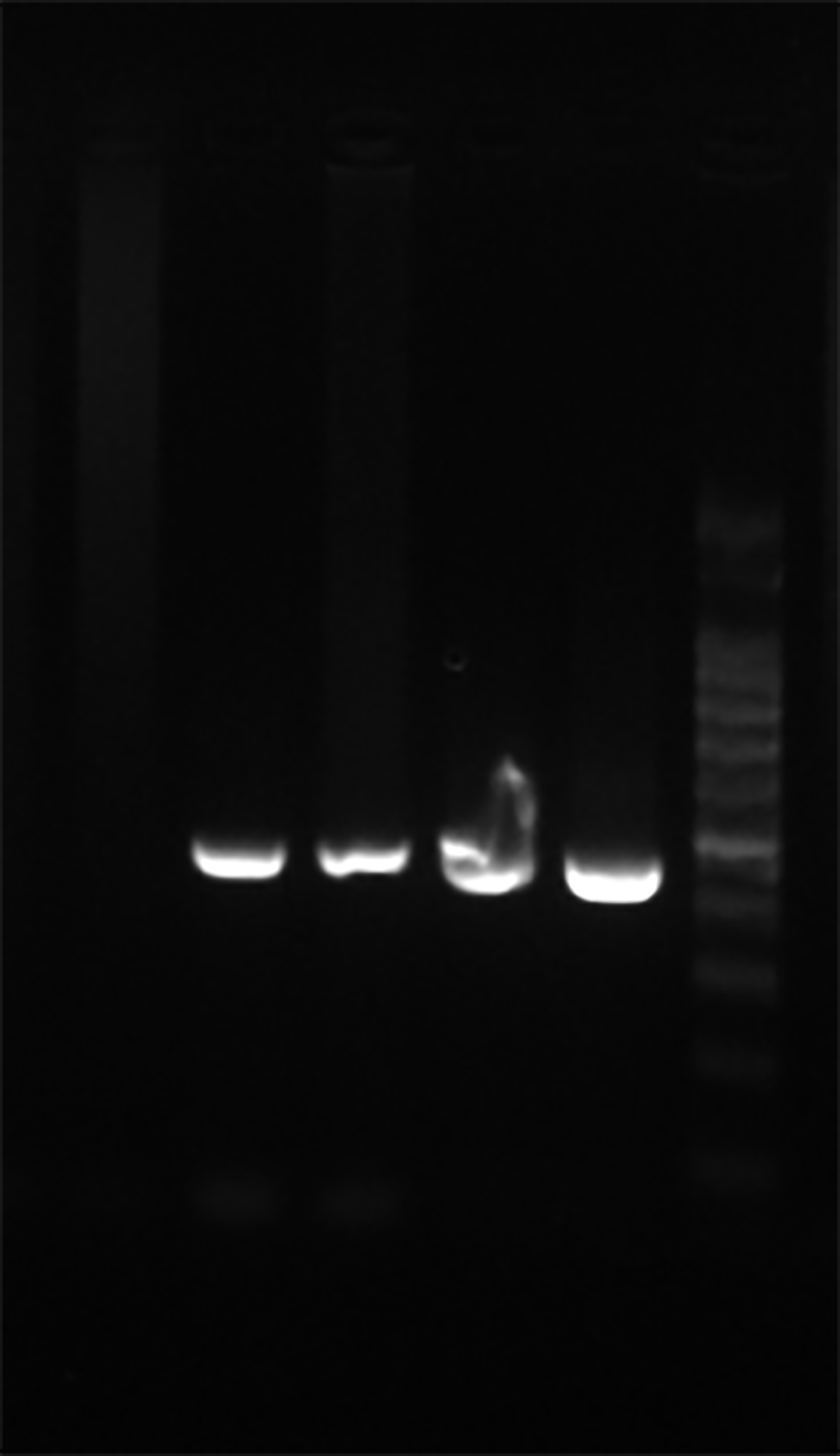

Supplement: Supplementary file 4 — Supplementary Information 4. [file 41598_2022_16196_MOESM4_ESM.zip › G. duodenalis bg gene.tif]

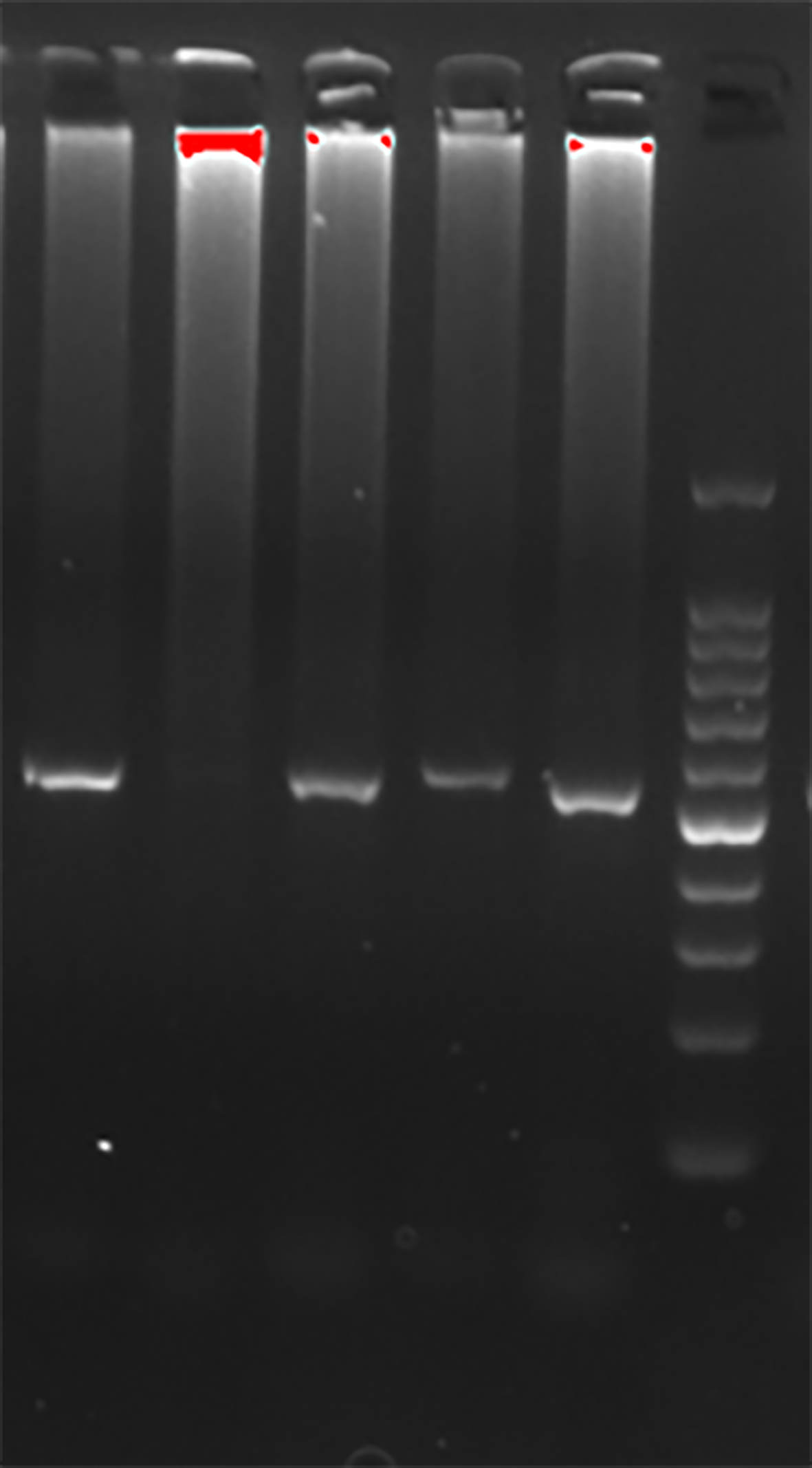

Supplement: Supplementary file 4 — Supplementary Information 4. [file 41598_2022_16196_MOESM4_ESM.zip › G. duodenalis gdh gene.tif]
