# Supplementary material for: Genotyping and subtyping of Cryptosporidium spp. and Giardia duodenalis isolates from two wild rodent species in Gansu Province, China
Source: Sci Rep. 2022 Jul 16;12:12178. doi: 10.1038/s41598-022-16196-1 (PMC9288474; doi:10.1038/s41598-022-16196-1)
Supplement: Supplementary file 3 — Supplementary Information 3. [file 41598_2022_16196_MOESM3_ESM.docx]

Table S1. Primers and reaction conditions in the characterization of the *SSU rRNA*, *HSP70*, and *actin* genes of *Cryptosporidium* spp. and the *bg*, *gdh*, *tpi* genes of *G. duodenalis.*

| Gene | Primer name | Primer sequence（5'-3'） | Amplicon size (bp) | Reaction condition | Reference |
| --- | --- | --- | --- | --- | --- |
| *SSU rRNA* | F1 | TTCTAGAGCTAATACATGCG | 830 | 94°C for 5 min; 35 cycles of 94°C for 45 s, 55°C for 45 s, and 72°C for 1 min; 72°C for 10 min | [22] |
|  | R1 | CCCATTTCCTTCGAAACAGGA |  |  |  |
|  | F2 | GGAAGGGTTGTATTTATTAGATAAAG |  |  |  |
|  | R2 | AAGGAGTAAGGAACAACCTCCA |  |  |  |
| *actin* | Act-F1 | ATG(A/G)G(A/T)GAAGAAG(A/T)A(A/G)(C/T)(A/T)CAAGC | 1066 | 94°C for 5 min; 35 cycles of 94°C for 45 s, 50°C for 45 s, and 72°C for 1 min; 72°C for 10 min | [61] |
|  | Act-R1 | AGAA(G/A)CA(C/T)TTTCTGTG(T/G)ACAAT |  |  |  |
|  | Act-F2 | CAAGC(A/T)TT(G/A)GTTGTTGA(T/C)AA |  |  |  |
|  | Act-R2 | TTTCTGTG(T/G)ACAAT(A/T)(G/C)(A/T)TGG |  |  |  |
| *HSP 70* | HSP-F1 | \| ATGTCTGAAGGTCCAGCTATTGGTATTGA \| \| --- \| | 1950 | 94°C for 5 min; 35 cycles of 94°C for 45 s, 55°C for 45 s, and 72°C for 1 min; 72°C for 10 min | [62] |
|  | HSP-R1 | TTAGTCGACCTCTTCAACAGTTGG |  |  |  |
|  | HSP-F2 | TA/CTTCATG/CTGTTGGTGTATGGAGAAA |  |  |  |
|  | HSP-R2 | CAACAGTTGGACCATTAGATCC |  |  |  |
| *gp60* | F1 | ATAGTCTCCGCTGTATTC | 850 | 94°C for 5 min; 35 cycles of 94°C for 45 s, 50°C for 45 s, and 72°C for 1 min; 72°C for 10 min | [23] |
|  | R1 | GGAAGGAACGATGTATCT |  |  |  |
|  | F2 | TCCGCTGTATTCTCAGCC |  |  |  |
|  | R2 | GCAGAGGAACCAGCATC |  |  |  |
| *bg* | BG-newF1 | AAGCCCGACGACCTCACCCGCAGTGC | 500 | 94°C for 1 min; 35 cycles of 94°C for 50 s, 56°C for 30 s, and 72°C for 1min; 72°C for 1 min | [24] |
|  | BG-newR1 | GAGGCCGCCCTGGATCTTCGAGACGAC |  |  |  |
|  | BG-newF2 | GAACGAACGAGATCGAGGTCCG |  |  |  |
|  | BG-newR2 | CTCGACGAGCTTCGTGTT |  |  |  |
| *gdh* | GDHF3 | TTCCGTRTYCAGTACAACTC | 530 | 94°C for 1 min; 35 cycles of 94°C for 50 s, 57.5°C for 30 s, and 72°C for 1min; 72°C for 1 min | [25] |
|  | GDHR3 | ACCTCGTTCTGRGTGGCGCA |  |  |  |
|  | GDHF4 | ATGACYGAGCTYCAGAGGCACGT |  |  |  |
|  | GDHR4 | GTGGCGCARGGCATGATGCA |  |  |  |
| *tpi* | TPI-F1 | AAATIATGCCTGCTCGTCG | 530 | 94°C for 1 min; 35 cycles of 94°C for 50 s, 56°C for 30 s, and 72°C for 1min; 72°C for 1 min | [26] |
|  | TPI-R1 | CAAACCTTITCCGCAAACC |  |  |  |
|  | TPI-F2 | CCCTTCATCGGIGGTAACTT |  |  |  |
|  | TPI-R2 | GTGGCCACCACICCCGTGCC |  |  |  |
